# Status Quo in the Liposome-Based Therapeutic Strategies Against Glioblastoma: “Targeting the Tumor and Tumor Microenvironment”

**DOI:** 10.3390/ijms252011271

**Published:** 2024-10-19

**Authors:** Mohd Haseeb, Imran Khan, Zeynep Kartal, Sadaf Mahfooz, Mustafa Aziz Hatiboglu

**Affiliations:** 1 Department of Molecular Biology, Beykoz Institute of Life Sciences and Biotechnology, Bezmialem Vakif University, Yalıköy St., Beykoz, 34820 Istanbul, Turkey; mohd.haseeb@bezmialem.edu.tr (M.H.); smahfooz@unmc.edu (S.M.); 2 Department of Biochemistry and Molecular Biology, University of Nebraska Medical Center, Omaha, NE 68198, USA; 3 Department of Radiation Oncology, College of Medicine, University of Nebraska Medical Center, Omaha, NE 68198, USA; 4 Department of Neurosurgery, Bezmialem Vakif University Medical School, Vatan Street, Fatih, 34093 Istanbul, Turkey

**Keywords:** liposomes, glioblastoma, microglia, TME, temozolomide, nanoparticles

## Abstract

Glioblastoma is the most aggressive and fatal brain cancer, characterized by a high growth rate, invasiveness, and treatment resistance. The presence of the blood–brain barrier (BBB) and blood–brain tumor barrier (BBTB) poses a challenging task for chemotherapeutics, resulting in low efficacy, bioavailability, and increased dose-associated side effects. Despite the rigorous treatment strategies, including surgical resection, radiotherapy, and adjuvant chemotherapy with temozolomide, overall survival remains poor. The failure of current chemotherapeutics and other treatment regimens in glioblastoma necessitates the development of new drug delivery methodologies to precisely and efficiently target glioblastoma. Nanoparticle-based drug delivery systems offer a better therapeutic option in glioblastoma, considering their small size, ease of diffusion, and ability to cross the BBB. Liposomes are a specific category of nanoparticles made up of fatty acids. Furthermore, liposomes can be surface-modified to target a particular receptor and are nontoxic. This review discusses various methods of liposome modification for active/directed targeting and various liposome-based therapeutic approaches in the delivery of current chemotherapeutic drugs and nucleic acids in targeting the glioblastoma and tumor microenvironment.

## 1. Introduction

Glioblastoma is one of the most aggressive and fatal cancer types in the central nervous system [1,2]. A rapid growth rate, high invasion capacity, and resistance to treatment are characteristic of glioblastomas. In recent years, studies have reported a steady increase in gliomas [2]. According to statistics, malignant gliomas are responsible for 2.5% of the global cancer death rate [3,4]. Depending on the report and region, there is substantial variation in the global incidence of glioblastoma, from 0.59 cases to 5 cases per 100,000 person-years. Recent data shows the reporting of an average of 12,000 cases per year in the United States alone, contributing to nearly 16% of all primary brain and central nervous system (CNS) malignancies [5].

Based on recent transcriptomic studies of tumors, glioblastoma has been divided into three subtypes: proneural, classical, and mesenchymal [6,7]. The mesenchymal subcategory of glioblastoma exhibits the worst form with poor prognosis, underscoring the significance of molecular variations in a single type of cancer. The standard treatment regimen for glioblastoma includes surgical resection, radiotherapy, and adjuvant chemotherapy with temozolomide (TMZ). Following maximal safe resection, adjuvant involved-field radiotherapy (RT) with concurrent (75 mg/m^2^/day) and post-RT adjuvant (150–200 mg/m^2^/day for 5 days in each 28 day-cycles) TMZ is the standard treatment recommended for patients with newly diagnosed glioblastoma based on the results of the phase III, randomized EORTC-NCIC study [8]. RT is the mainstay of the treatment in patients with glioblastoma and is commonly given in 30 fractions with a dose of 2Gy/fraction. RT has provided moderate survival benefits in patients with no significant damage to neuro-recognition and the quality of life. There are also some emerging radiation therapy options such as single fraction and hypo-fraction radiation treatments. Hypofractionated radiotherapy (a higher dose of radiation per fraction over fewer treatments) has been used in elderly patients (≥70 years) with reduced performance status, neurological deficits, and severe medical comorbidities [9,10]. Moreover, in a recent study, we reported that radiation treatment with hypofractionated treatment using Gamma Knife radiosurgery provided a survival benefit and improved local tumor control compared to single-fraction treatment in patients with recurrent glioblastoma [11]. Despite the presence of such a rigorous treatment regimen, the overall survival is still poor, with a median survival of approximately 15 months from the first diagnosis [12].

Current chemotherapy regimens have limitations and suffer from a lack of specificity, limited and poor bioavailability, low efficacy, poor solubility, and nonspecific interactions, often failing to eliminate the tumor [13]. Additionally, the presence of the blood-brain barrier (BBB) and blood-brain tumor barrier (BBTB) imposes further difficulties for the use of chemotherapeutics. Owing to these limitations of current treatment modalities and the challenges enforced by BBB and BBTB, there has been an increase in more specific and specialized drug delivery systems to effectively cross these barriers and to deliver the drug to its target [14,15].

Over the past decade, nanoparticles (NPs) have attracted considerable attention from biomedical researchers due to their unique properties. These nano-sized particles have a small size with a high surface area to volume ratio and exhibit many significant properties, such as optical, magnetic, and photoelectric properties, which can be exploited further for customized biomedical applications [16]. Additionally, NPs exhibit antimicrobial, cytotoxic, and anticancer properties, depending on their size and shape [17]. Such properties encourage their application in clinical research and drug delivery. Furthermore, the ease of synthesis and surface modification/functionalization by utilizing different conjugation strategies can be used to achieve specific targeting. Nano-based drug delivery approaches rely on engineered NPs for the cargo or delivery of chemotherapeutics to the targets. The utilization of such nanoscale carriers offers the selective advantage of site-specific delivery, enhanced bioavailability, increased solubility, and reduction of dose-dependent toxicity of the drugs.

Various chemotherapeutics, antibodies, tumor-derived antigens, and receptor-specific ligands have been conjugated and directed against multiple targets [18,19,20,21]. Their miniaturized size permits these NPs to overcome diffusional limitations and penetrate cellular barriers with ease. In addition, the high surface area to volume ratio aids in high drug loading and maximizes the interaction with the target [21]. Many types of NPs, varying in their shape, size, and synthesis routes, find their application in biological/biomedical research [22]. Inorganic metal NPs (gold, silver, iron, zinc, etc.), carbon dots, carbon nanotubes, micelles, and liposomes are popular categories of NPs in biomedical applications [23]. The presence of the BBB is one of the most significant obstacles in the delivery of the drug to the target in the CNS. The smaller size allows these particles to breach and penetrate the BBB, which permits the delivery of chemotherapeutics to brain tumors [24]. The ability of these nanostructured molecules to cross the BBB and deliver drugs or payloads efficiently without significantly altering or compromising the permeability of the BBB is well established. NPs have revolutionized the field of drug delivery systems [25,26]. Apart from BBB penetration, NP-based drug delivery systems offer a significant improvement in the pharmacodynamics and pharmacokinetics of drugs, enhanced permeability and retention (EPR), and bioaccumulation within the cancer cells [27]. Physicochemical attributes of NPs, such as shape, size, surface hydrophobicity, surface charge as well as the properties of the plasma membrane, impact the cellular permeability or cellular uptake of NPs; these particles can enter the cells actively or passively [28]. Additionally, it has been reported that NPs use another strategy of cellular entry by taking advantage of low-intensity endocytosis by endothelial cells to engulf the drug-conjugated/drug-loaded NPs and deliver the drug across the BBB to target cells [29]. Despite the ability of a reticuloendothelial system (RES) to prevent damage in normal healthy cells and promote their clearance out of the body, the majority of metal or inorganic NPs exhibit toxicity in a dose-dependent manner, which is of great concern while designing a NP-based drug delivery system for human use [30].

Considering the toxicity of nanocarriers, liposomes, which are among the commonly used nanocarriers, offer one of the safest approaches for delivering cargo to the target, and have been utilized to deliver drugs, vaccines, nucleic acids, and proteins [31,32]. These lipid-based carriers were first discovered in 1960 and are composed of fatty acids, phospholipids, and cholesterol; they can be further decorated with various targeting molecules [17,33]. Liposomes exhibit excellent biocompatibility, biodegradability, and negligible toxicity [34,35]. Due to these advantages, liposomes have been under investigation extensively for the delivery of drugs for glioblastoma treatment [36].

Our main focus in this review is to explore the role of liposomes in the targeted delivery of therapeutic payloads, including chemotherapeutic drugs, nucleic acids, and targeting the tumor microenvironment (TME) and microglia to combat glioblastoma.

## 2. Liposomes: Background and Overview

Liposomes are small, spherical, and synthetic molecules in the range of nanometers to a few micrometers [37]. These synthetic vesicles are composed of a phospholipid bilayer, encapsulating an aqueous core, and closely resemble cellular structures. Phospholipids are amphipathic molecules that have polar, hydrophilic heads and nonpolar/lipophilic tails [38]. When these lipids are suspended in water, they spontaneously make a tail-to-tail arrangement to minimize water interaction and, thereby, encapsulate an aqueous core, where the polar heads protrude toward the aqueous surface, and the nonpolar, hydrophobic tail face inward, away from water interaction. This particular assembly of phospholipids makes these structures amphipathic, where they have both polarity and non-polarity. They can house polar/hydrophilic substances on their hydrophilic side, whereas their nonpolar side can be exploited for hydrophobic molecules.

Both natural or synthetic phospholipids can be used to construct liposomes. These include phosphatidylcholine, phosphatidylethanolamine, phosphatidylserine, and phosphatidylglycerol [39]. In the early stages of their discovery, liposomes were mainly synthesized using only natural lipids, but the scenario has gone through a remarkable change, as now, liposomes are synthesized via the utilization of natural, synthetic anionic, and cationic lipid precursors. Even now, their surface properties have been modified by adding surfactants to alter their composition to enhance stability, increase circulation in vivo, and improve retention, permeability, and drug/cargo loading capacity.

Furthermore, the liposomal surface can be modified with ligands specific for particular receptors on target cells to deliver chemotherapeutic payloads to the target through active targeting. The surface functionalization of liposomes can be attained by utilizing a range of specific molecules, including proteins, peptides, carbohydrates, aptamers, monoclonal antibodies, NPs, or other ligand molecules of choice. Functionalized liposomes specifically recognize and bind to target receptors via ligand–receptor interactions, are internalized via endocytosis, and deliver their payload to target cells [40]. This mode of targeted delivery reduces the chances of non-specific interactions and enhances the efficacy of therapeutics under consideration. Such strategies are highly significant in cancer targeting, as certain receptors are found to be overexpressed on some tumors. In modern clinical research and drug development, such synthesized liposomes are extensively investigated for targeted drug delivery approaches due to their distinguished properties, such as low immunogenicity, high biocompatibility, and low toxicity in biological systems.

Depending on the synthetic routes and the size of precursor molecules, liposomes can have variable sizes and shapes, ranging from a few to a hundred nanometers or even to micrometers. Liposomes can be further classified broadly into two main groups: (1) classical or unmodified liposomes; and (2) modified/functionalized or engineered liposomes.

### 2.1. Unmodified/Classical Liposomes: (Native/Unaltered)

**Figure 1:**
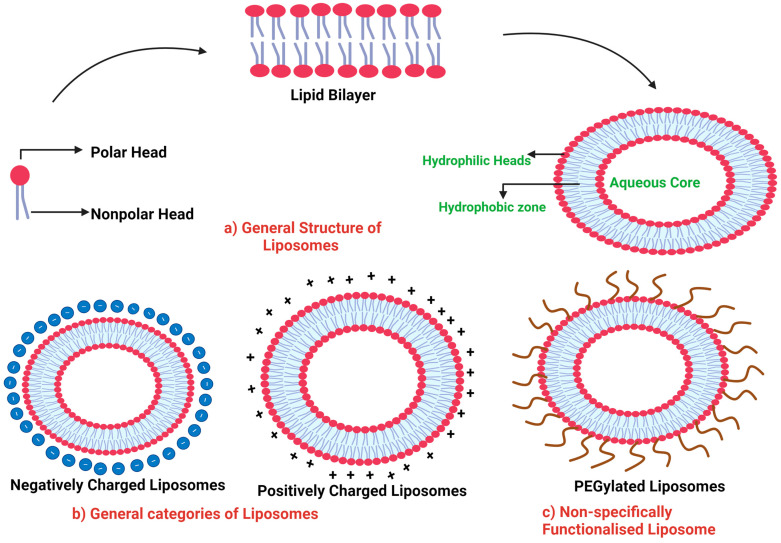
(**a**) Representing the basic structure of a liposome, (**b**) showing the charged liposomes, and (**c**) PEGylated liposomes.

Unmodified or classical liposomal assemblies rely on using phospholipids without any further modification. Such liposomes are also called non-engineered/native or classical liposomes. Based on overall charge, due to phospholipid content and subtypes, unmodified liposomes can be further classified as (a) uncharged liposomes and (b) charged liposomes. Charged liposomes can be either cationic or anionic (Figure 1).

The net charge on the surface or cellular microenvironment dictates the choice of the charge type on liposomes [41]. The exact choice of liposomal type will depend on the area or environment being targeted. For example, nucleic acids, being negatively charged, can be effectively delivered via cationic liposomes due to strong electrostatic interactions, resulting in structures called lipoplexes (lipid complexes) [42]. Such lipoplexes provide stability to genetic material and protect nucleic acids from enzymatic cleavage. The use of cationic liposomes in the delivery of therapeutics to the glioblastoma is useful due to the negative charge of the BBB, which may favor electrostatic interactions, resulting in enhanced uptake via adsorption-mediated endocytosis [43]. However, this approach is problematic on a few occasions, as cationic liposomes may be taken up by other cells or their coating with free circulating plasma proteins, due to electrostatic interactions, may enable immune clearance. To overcome this limitation, pre-coating liposomes with plasma proteins of choice can be an advisable strategy [44].

Conventional or unmodified liposomes are highly biodegradable and biocompatible, and they exert a low risk of toxicity in vivo and in vitro, finding extensive applications as drug carriers in a vast array of currently approved and under-approval drugs. Although these unmodified or classical liposomes have numerous advantages, they may exhibit multiple drawbacks depending on the route of administration. For example, intravenous (IV) administration of liposomes results in serum protein adsorption, leading to clearance by the hepatic RES in the liver, whereas administration via the oral route subjects the liposome to harsh gastrointestinal conditions, such as the high acidity of the stomach, bile salts, and lipases. In the case of glioblastoma, liposomal targeting strategies face even more challenges due to the BBB separating the CNS from the bloodstream, as well as efflux transporter proteins, which expel the payload out of the cells [45,46].

### 2.2. Modified/Engineered or Functionalized Liposomes

Engineered liposomes are those that have undergone modifications or significant alterations to their surface to overcome the limitations of conventional liposomes to help them penetrate barriers across biological systems. The liposomal surface can be modified or altered to generate engineered or functionalized liposomes. Such modifications can help overcome the limitations associated with unmodified or conventional liposomes and further enhance the ability of liposomes to cross biological barriers [36].

Furthermore, liposomes can be functionalized via the incorporation of specific ligands/proteins/engineered molecules/antibodies to selectively target specific receptors on target cells.

PEGylation is one of the most commonly employed methods, utilizing polyethylene glycol (PEG) polymers to covalently modify the surface and enhance the blood dynamics of liposomes [47]. It involves the conjugation of liposomes with PEG molecules via covalent linkage. This enhances stability, circulation ability, and half-life in vivo. It has been shown that PEGylation can prevent opsonin adsorption on the surface of administered liposomes, thereby preventing the phagocytic clearance of liposomes by the mononuclear phagocytes in the spleen and liver. This enhances the circulation time of liposomes, along with the encapsulated therapeutic payload, in the bloodstream [48].

Gref et al. demonstrated in the early 1990s that the circulation time of PEGylated NPs depends on the molecular weight (MW) of PEGs: the larger the MW, the longer the lifetime of the final nanoparticle [49]. A study conducted by Chow et al. on PEGylated liposomes led to similar findings, showing that a higher concentration of PEG (6 mol%) improved the pharmacokinetic properties of the final liposomes compared to a lower concentration of PEG (0.9 mol%) [50]. The enhanced accumulation of these long-circulating carrier systems in tumor cells was associated with increased leakiness of blood vessels and reduced lymphatic drainage in tumors, referred to as the enhanced permeability and retention (EPR) effect [27].

Despite exhibiting several advantages, PEGylation has significant disadvantages, as PEG can hinder the process of liposomal uptake through transcytosis or endocytosis, resulting in poor uptake by tumor cells. This may also inhibit the target–ligand interactions between ligands on liposomes and their complementary receptors on the target cancer cells, causing poor delivery and release of drugs [51]. This can result in an overall reduction in efficacy, bioavailability, and effective targeting.

Another popular strategy for the surface modification of liposomes is the utilization of polysaccharides to address the concerns regarding their bioavailability, biodegradation, and biocompatibility of liposomes [52]. Chitosan is one such polysaccharide molecule that is being used to produce another modified category called chitosanylated liposomes. Chitosan is a deacetylated derivative of chitin. It is a linear cationic molecule composed of D-glucosamine and N-Acetyl-D-glucosamine residues joined through a glycosidic β (1 → 4) linkage. Chitosan has a mucoadhesive nature, which escalates the penetrability of modified liposomes, especially those delivered via mucosal surfaces, such as the nasal and intestinal routes. The extent of deacetylation and the average molecular mass are two essential properties in terms of the quality of chitosanylated liposomes. The extent of deacetylation depends on the level of glucosamine residues and falls anywhere in the range of 30–95%, with an average molecular weight of 126–1000 kDa [53]. Apart from chitosan, other carbohydrates, including glucose, can also be utilized for liposome functionalization. Zhang et al. [43] utilized glucose to target glioblastoma via over-expressed GLUT1 receptors on cancer cells and observed an enhanced uptake of liposomal payloads. Glucose-mediated functionalization of liposomes has also been done in combination with biotin and vitamin C for better internalization and uptake [54,55]. The monosaccharide mannose, which is a C-2 epimer of glucose, has also been used to functionalize liposomes for enhanced circulation time and better uptake by target cells [56]. The detailed mechanisms and effects of such functionalization approaches, along with the respective payloads, are discussed in later subsections.

### 2.3. Direct Targeting Liposomes Against Glioblastoma and Methods of Functionalization

Delivering drugs to cancer cells, especially via targeting specific and unique receptors, is one of the major challenges in glioblastoma therapy. The presence of the BBB, suppressive TME, and the lack of specific over-expressed and unique targeting moieties on the tumor cells have presented a mammoth task in glioblastoma therapeutic research and development. Selective targeting can minimize the drug’s dose-dependent side effects, enhance their bioavailability and pharmacological effects, and enable the development of more specific and personalized chemotherapeutic approaches. To achieve this, merely modifying the surface via nonspecific mechanisms such as PEGylation and chitosanylation is not sufficient; rather, a more directed approach by incorporating a targeting ligand in liposomes, specific to particular receptors, unique or overexpressed on target cells, is essential. This process, called targeting, is a complex mechanism and involves modifications/functionalization by incorporating specific “targeting” molecules.

A wide range of ligands, including proteins or peptides, carbohydrates, immunoglobulins, and aptamers, can be covalently or non-covalently conjugated to the liposomal surface.

**Figure 2:**
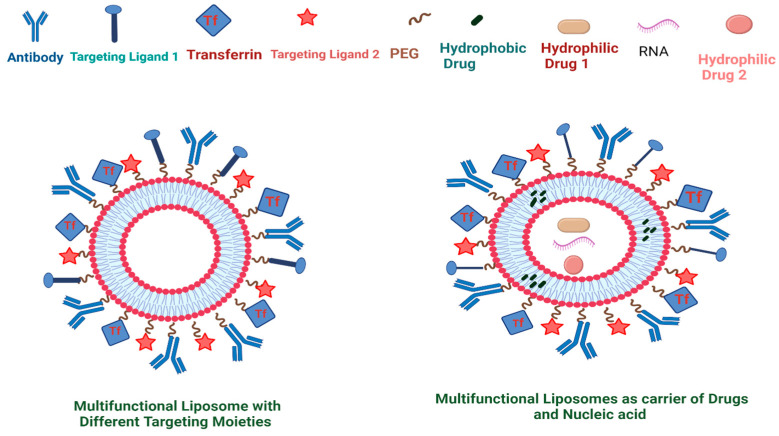
Typical representation of a multifunctional liposome, modified with different targeting groups/ligands/peptides or antibodies, and loaded with therapeutics of interest.

This conjugation process exploits a diverse range of molecules with specific functional groups. A schematic representation of a multifunctional target-specific liposome is shown in Figure 2. Covalent conjugation can be accomplished through different coupling mechanisms, such as carboxamide linkage, thioether linkage, amide bonds, disulfide linkage, and hydrazone bonds [57]. The carboxamide conjugation involves the exploitation of an anchor molecule, with carboxylic acid as a terminal group. The coupling reaction produces an acyl amino ester in the presence of carbodiimides, which ultimately reacts with the primary amine of the ligand to form an amide bond [16]. Liposome functionalization with thioether involves the reaction of the maleimide group of certain phospholipids with the thiol group of ligands, such as the Fab regions of immunoglobulins (as an example), to form stable covalent linkage [58]. The ligand’s amine groups are transformed into free thiols (sulfhydryl group) with the help of a chemical called Traut’s reagent, which can react further with the maleimide group on the liposomal surface.

Disulfide bridges, involving a reaction between thiol groups (-SH) present on the substrate surface and thiol groups of the ligand, are an additional method to chemically attach a ligand to the liposomal surface. However, this method may promote the homocoupling of thiols, where disulfide bridge formation can occur between two same type of molecules containing thiols, resulting in decreased sensitivity and selectivity. Additionally, disulfide bond cleavage via reducing agents or enzymes can serve as an advantage for particular applications [59].

Another functionalization strategy is the utilization of a hydrazone bond to couple antibodies to carbohydrate ligands by adding hydrazide groups to the surface of the liposome. The oxidation of carbohydrate groups can normally be achieved via exploiting sodium periodate or galactose oxidase. Post-oxidation, the oxidized antibodies can be linked to a bilayer of lipids possessing a hydrazide-hydrophobic anchor, such as lauric acid hydrazide or similar groups present at the other end of the PEG chains of sterically stabilized liposomes [60].

Liposomes can be functionalized with target-specific biomolecules through non-covalent interactions, such as electrostatic attractions, van der Waals forces, and hydrogen bonding; although these forces are individually weak, when taken together, they form strong complexes [60]. Despite its proven efficiency, a lack of control in the orientation of target ligands on the surface is one of the most significant challenges that may influence overall stability and functionality. As such, non-covalent interactions are affected by changes in the physicochemical conditions of the reaction environment, such as ionic strength, pH, temperature, or the isoelectric point; any change herein can alter the electrostatic or non-covalent interactions. Let us understand this from the perspective of a tertiary protein: The structure and orientation play a very important role to play in the functionality of a protein molecule, and even a slight change in the structure of the tertiary protein attached to the liposomal surface via weak hydrophobic interaction will have a considerable impact on its biological activity [34].

### 2.4. Tumor Microenvironment Targeting via Functionalized Liposomes in Glioblastoma

The tumor’s immediate surroundings, referred to as the tumor microenvironment, are extremely complex due to cellular heterogeneity. Within the TME, tumor cells coexist and maintain a subtle interaction with a large plethora of cells from the immune system (B and T lymphocytes, natural killer cells (NKCs), macrophages, dendritic cells, polymorph nuclear cells, and mast cells) and various non-immune cells, such as endothelial cells and stromal cells [61]. This interaction and co-existence of tumor cells with other cell types in the TME determine the natural history of the tumor. The tumor’s fate, invasiveness, and ability to metastasize are primarily determined by immune cells within the TME [62]. Immune cell infiltration into tumors and their composition within the TME are closely linked to clinical outcomes in cancer patients. Considering the above, the TME could be an additional target for liposome-based tumor-specific active targeting strategies, apart from approaches targeting oncogenic surface receptors on cancer cells. TME and microglial targeting are discussed in detail in a later section of this review.

There are a variety of biomarkers specifically associated with the TME, such as enzymes, extracellular matrix components, and tumor-specific pathophysiological circumstances, which can be targeted using liposome-based nanomedicine [63]. Liposomes have the potential to block tumor diffusion by targeting the tumor vasculature [40]. TME targeting via liposomes is broadly discussed in Section 4 of this review.

### 2.5. Stimuli Sensitive Liposomes

Apart from ligand-based, site-specific surface modifications, liposomal surfaces can be modified with chemical moieties, resulting in a new category of liposomes called smart or stimuli-responsive liposomes, which respond differently to various stimuli or physiological conditions prevalent at the tumor site or in the tumor microenvironment.

Site-specific targeting can also be achieved by attaching sensitive ligands onto liposomal surfaces via surface functionalization. Liposomal surfaces can also be engineered via some chemicals or specific functional groups that are sensitive and responsive to particular changes or stimuli at the site of delivery [64,65]. Liposomal content is released at the site of delivery under the effect of certain changes enforced by ligands or stimuli. Stimuli influencing the release of liposomal payloads can be intrinsic or extrinsic. Various internal stimuli, such as pH gradients, enzymes, temperature gradients, and redox potential, could be exploited for targeted release. Alternatively, factors such as temperature, ultrasound, light, or magnetic fields can be utilized as external factors for stimuli-sensitive liposomes [66,67]. Such stimuli-sensitive liposomes are highly beneficial for the delivery of chemotherapeutics with low bioavailability and poor biodistribution, as these result in the enhanced accumulation of drugs at the tumor site. Zhou et al. [68] developed pH-redox-sensitive, cascade-targeted liposomes by modifying the surface with glucose and TPP to carry a dual payload of doxorubicin prodrug and lonidamine against glioma. The results demonstrated a successful release of the payload, as the acidic environment in the glioma played the role of the stimulus, triggering the release of drug molecules from the liposomes. The acidic environment in the TME was also exploited by Torres et al. to trigger drug release through ion channel-based, pH-sensitive liposomes [69]. In another study, thermosensitive magnetic liposomes (TML) were developed to encapsulate a dual payload of camptosar (CPT-11) and citric acid-functionalized magnetic nanoparticles. Cetuximab was used to modify the TML surface further to target overexpressed epidermal growth factor receptors on the target cells specifically. A high alternating magnetic field was used to increase the temperature to promote drug release after treatment with TML in vitro. A similar therapeutic effect was also observed in an in vivo orthotropic xenograft mouse tumor model [70].

## 3. Liposome-Based Therapeutics in Combating Glioblastoma

### 3.1. Liposomes in the Delivery of Temozolomide against Glioblastoma

TMZ is an alkylating agent administered orally and causes DNA damage in the target cells by selectively transferring alkyl groups to guanine nucleotide bases, resulting in DNA damage-based apoptosis. It was first synthesized in 1984 by Stevens [71] and added to the standard of care in glioblastoma therapy after a successful clinical trial in 2005, resulting in an overall survival benefit of 7.6 months [8]. Additional clinical studies validated that simultaneous radiotherapy and TMZ treatment, followed by adjuvant TMZ, resulted in remarkable enhancements in the median survival of the patients from 12.1 months (with chemotherapy alone) to 14.6 months. Additionally, TMZ increased the 2-year survival rate from 10.4% to 26.5%

TMZ, in its native form, is therapeutically inactive, and its metabolism requires a pH-dependent reaction in a physiological medium, where it becomes hydrolyzed into its active form: 5-(3-methyltriazen-1-yl) imidazole-4-carboxamide (MTIC). MTIC is a polar molecule and becomes further catabolized to 5-aminoimidazole-4-carboxamide (AIC), and methyldiazonium cations and AIC are excreted via the kidney. This methyldiazonium cation is a methylating agent responsible for DNA damage via methyl group transfer to the N^7^ of guanine in guanine-rich regions. Its ability to methylate O^6^ guanine and N^3^ adenine has also been demonstrated [72,73,74]. It must also be noted that its activity to induce apoptosis via methylation of nitrogenous bases and DNA damage is only effective in the absence of cell repair mechanisms [75].

However, the therapeutic potential of TMZ in glioblastoma treatment is not optimal due to its inability to effectively penetrate the BBB, as well as its very short half-life in vivo (~2 h). These limitations push for higher doses of TMZ for effective treatment, resulting in severe dose-dependent side effects, systemic toxicity, drug resistance, etc. [76]. Under these circumstances, it is highly imperative to devise strategies, such as nanoparticle-based encapsulations or formulations, that will not only prevent non-specific interactions but also enhance efficacy, bioavailability, and reduce toxicity to normal cells. Owing to this, there has been a surge in nanoparticle-based drug delivery applications for glioblastoma. Nanomedicine, especially liposome-based therapies, can help overcome these problems. The encapsulation of TMZ offers different advantages, including (i) the improvement of solubility and stability, (ii) better brain accumulation, (iii) a reduction in high dosage administration, and (iv) a decrease in side effects [77].

#### 3.1.1. In Vivo Studies

According to available literary evidence, Ab initio (Primus semper) research reporting of the liposome encapsulation of TMZ emerged in 2009 and 2010 [78,79]. Interestingly, the breakthrough report regarding liposome–TMZ encapsulation for glioblastoma was indexed in 2015, where researchers analyzed the biodistribution and pharmacokinetic profiles of administered liposomes in healthy mice and rabbits, demonstrating the prolonged half-life of TMZ encapsulated in liposomes [73]. Additionally, the concentration of encapsulated TMZ was reported to be higher in the brains of experimental animals. The accumulation of TMZ-liposomes in the brain was explained by the presence of sorbitol in the liposome composition, which prevented the rejection of the liposomes by the reticuloendothelial system (RES). Furthermore, there was less encapsulated TMZ accumulation in the lungs and heart, indicating limited side effects in these organs. Nonetheless, the liver, kidney, and spleen were reported to have a higher encapsulated TMZ concentration, indicating concern about this delivery strategy. Such an organ-dependent accumulation can be attributed to the presence of macrophages, which may take up encapsulated TMZ-–liposome assemblies. Moreover, plasma protein markings on the liposomal surface can enable their clearance via the RES, leading to clearance from the liver and spleen.

To overcome these limitations, the incorporation of PEG on the surface of liposomes, called PEGylation, seems to be a comparatively more effective strategy, as demonstrated by some studies. A study conducted in this regard has shown that the use of PEGylated liposomes resulted in the enhancement of the concentration of TMZ in plasma and the brain. Additionally, they demonstrated a delay in the clearance of PEGylated liposome–TMZ assemblies, signifying the role of PEGylation in combating RES-mediated clearance [80].

Delivery of encapsulated TMZ in PEGylated liposomes directly to the brain is another effective method for enhancing therapeutic potential while significantly minimizing non-specific interactions accompanying intravenous administration. In agreement, Lin et al. used convection-enhanced delivery (CED) to directly deliver the PEGylated liposomal TMZ (8.7 mg/mL TMZ, dipalmitoylphosphatidylcholine—DPPC, cholesterol) to the brain of U87G tumor-bearing Nu/Nu mice [81]. This combined liposome–TMZ assembly with CED allowed for the delivery of a low but sufficient quantity of TMZ without any significant systemic toxicity. However, there was no significant change in survival time between mice receiving 5 µL (0.0435 mg/mouse) of liposomal TMZ via CED and those in the control group. Moreover, repeated dosing of 5 µL (0.0435 mg/mouse) of the liposomal TMZ in the same mice after 7 days of the first administration resulted in an improved survival time of more than 70 days. When mice were administered 10 µL of liposomal TMZ, the survival time was again found to be similar to that of a single dose. However, this study failed to include a comparison with the survival rate of mice being administered TMZ-liposomes in a systemic fashion or with other similar studies, making it difficult to appreciate the obtained results at their real value. On the other hand, no systemic toxicity or brain damage was noticed after a three-day administration of the TMZ liposomal formulation. In another study, PEGylated liposomes composed of DSPC (1,2-distearoyl-sn-glycero-3-phosphocholine) and cholesterol were used to encapsulate TMZ at a concentration of 1.7 mg/mL and subsequently delivered via CED, showing a similar impact [82].

PEGylated liposomes made of soybean phosphatidylcholine (PC), dipalmitoyl phosphatidylcholine (DPPC), dipalmitoyl phosphatidylcholine-sodium (DPPG), egg lecithin, cholesterol, and 1,2-distearoyl-phosphatidylethanolamine-methyl-polyethyleneglycol conjugate-2000 (Na^+^ salt) have been used to encapsulate TMZ for glioblastoma [83]. The in vitro results were quite interesting, with prolonged TMZ release of up to 800 min from liposomal encapsulation in comparison to free TMZ, which was released in 90 min. Pharmacokinetic analysis conducted in female Sprague–Dawley rats demonstrated a decreased clearance of PEGylated liposomes encapsulating TMZ by 1.5-fold, in contrast to TMZ in its free form.

In a study by Ying Zhang et al., a new strategy for targeting glioblastoma via the GLUT1 receptor was used [43]. The study utilized liposomes functionalized with glucose (gLTP) to co-deliver encapsulated temozolomide and pro-apoptotic peptide (PAP) for enhanced efficacy in glioblastoma. The researchers demonstrated that the functionalized liposomes efficiently penetrated the BBB via glucose–GLUT1 interaction and subsequently release the payload of TMZ and PAP inside the target. The inclusion of PAP resulted in the sensitivity of glioblastoma cells to TMZ, as PAP has the potential to destroy mitochondria, resulting in depleted levels of ATP. Furthermore, the efficacy of gLTP-encapsulated TMZ-PAP was found to have the highest antitumor effect in comparison to TMZ-liposomes, PAP-liposomes alone, or a physical mixture of PAP and TMZ in a subcutaneous brain tumor model. Moreover, the same assembly was found to be effective in crossing and delivering the payloads to an aggressive intracranial tumor model.

Lam et al. developed a transferrin-conjugated liposomal model encapsulating TMZ and bromodomain inhibitor JQ1 as a dual payload for more effective glioblastoma targeting [84]. The rationale behind the inclusion of bromodomain inhibitor JQ1 was its ability to block protein interactions and induce apoptosis, as the bromodomain inhibitor JQ1 is an epigenetic drug that prevents interaction between transcriptional proteins. Moreover, it has a potential role in the inhibition of pathways for DNA damage repair, thereby enhancing the TMZ sensitivity of glioblastoma [85,86]. The authors demonstrated that transferrin functionalization enhanced effective delivery across the BBB in NCR nude mice and resulted in an increased accumulation of liposomal-drug assemblies in the endothelial walls of the brain. Results indicated the role of potential DNA damage and apoptosis, resulting in around a 2-fold (1.5–2 fold) reduction in tumor burden along with increased overall survival. This study signifies the use of transferrin for effective targeting of the BBB as well as the role of dual payloads in achieving more potent tumor cell death.

Delivering liposome-TMZ formulations across the BBB has always been challenging due to the obvious reasons discussed above. Keeping this in mind, a group used ultrasound-mediated BBB-opening technology to deliver liposomes encapsulating temozolomide in tumor-bearing rat models [87]. They synthesized cholesterol-embedded DPCC liposomes for the encapsulation of TMZ via a thin-film hydration technique and tested the resulting mixture against in vitro BBB models and in vivo animal models. The ultrasound irradiation-mediated delivery resulted in enhanced in vitro cytotoxicity and enhanced TMZ concentration distribution of in vivo anti-tumor efficacy in comparison to control groups.

To explore a more directed and efficient approach to targeting glioblastoma via TMZ-liposome assemblies, an ART-PC (artesunate-phosphatidylcholine) liposomal assembly was used by Ismail Muhammad et al. [57]. Artesunate is a derivative of artemisinin, a traditional Chinese natural medicine that exhibits strong antitumor activities and induces DNA damage via the generation of ROS [88]. This novel combination of artesunate-phosphatidylcholine liposomes packed with TMZ was able to efficiently co-deliver the combined payload of TMZ-ART to TMZ-resistant U251-TR in vivo as well as in vitro. Further analysis showed that the administered combination inhibited DNA repair, enhanced DNA damage via synergy, and augmented apoptotic cell death, thereby showing an overall improvement in glioblastoma sensitivity to TMZ. Moreover, it successfully resulted in the reduction of U251-TR glioma burden in animal models, enhanced its ability to penetrate the intracranial tumor, and improved overall survival in tumor-bearing mice.

A unique methodology comprising magnetic temperature-sensitive liposomes encapsulating temozolomide as a payload has been demonstrated by Yao et al. [89] for the targeted release of TMZ inside the tumor through a thermo-magnet-dependent mechanism. The authors synthesized “thermosensitive” liposomes using different combinations of DPPC, DSPC, and DSPE-mPEG2000 in molar ratios to incorporate magnetic NPs and TMZ. Subsequently, the release of encapsulated TMZ was triggered by Fe_3_O_4_ MNPs using an alternating magnetic field (AMF), causing a significant amount of oxidative damage to cells via ROS induction, leading to cell death in U87 and U251 cell lines. The authors believed the above combination was effective in inducing cell death via ROS-mediated pyroptosis, not apoptosis, as part of their experimental findings.

#### 3.1.2. In Vitro Studies

Encapsulated TMZ-liposomes have also been delivered via an in situ hydrogel model consisting of collagen and hyaluronic acid (HA). The TMZ-loaded liposomes were synthesized using a lipid combination consisting of HSPC/cholesterol/DSPE-PEG2000 in a fixed molar ratio via the thin-film method [90]. The liposome–hydrogel composite was obtained by transferring TMZ-liposomes to a collagen solution before the addition of other gel components, followed by incubation at 37 °C. The resulting composite was analyzed for its anti-tumor effect against a 3D spheroid glioblastoma model in vitro. The authors reported gelling of the composite within a minute at 37 °C, showing deep tumor penetration via sustained release and exhibiting substantial glioma cell growth. Although these results seemed to be significant, further evaluation of these composites in in vivo tumor models is warranted to assess their efficacy, stability, and bioavailability in complex and dynamic in vivo environments.

In another study aimed at targeting the BBB via TMZ-encapsulating liposomes, researchers synthesized a set of four cationic liposomes to exploit the targeting nature of the biomolecular corona (BC) layer, formed around cationic liposomes after their exposure to human plasma [91]. The biomolecular corona is the first layer that forms when these liposomes are introduced into the biological system. The authors used cationic lipids 1,2-dioleoyl-3-trimethylammonium-propane (DOTAP) and 3(-[*N*-(*N*′, *N*′-dimethylaminoethane)-carbamoyl]-cholesterol (DC-Chol) as well as neutral lipids dioleoylphosphatidylethanolamine (DOPE) and cholesterol for the synthesis of cationic liposomes. This work shows that the biomolecular corona was rich in BC fingerprints such as vitronectin, apo-lipoproteins, and vitamin K-dependent proteins, which have good efficiency in binding to various overexpressed receptors on the BBB, including class B scavenger receptors, type I receptors, and low-density lipoprotein receptors. The researchers further used human umbilical vein endothelial cells (HUVECs) as an in vitro model of the BBB to show the efficient uptake of synthesized biomolecular corona-cationic lipid assemblies (CL-BC) encapsulating TMZ. Furthermore, the formulation was tested against U87 MG cell lines and was reported to deliver the payload with comparatively higher efficiency than corona-free cationic liposomes. A summary of various studies reporting the use of liposomes in the delivery of TMZ has been provided in the Table 1.

### 3.2. Liposomes Encapsulating Doxorubicin as the Payload against Glioblastoma

Doxorubicin (DOX) is a topoisomerase II inhibitor that belongs to a category of drugs called anthracyclines (others include idarubicin and daunorubicin). Doxorubicin causes DNA damage via strand breaks and hampers the action of enzymes called topoisomerases, which are specific to DNA replication and transcription, thereby preventing tumor growth [92]. It is one of the most frequently used anticancer drugs and has been used to successfully treat various carcinomas, such as leukemias, aggressive lymphomas, osteosarcomas, and breast cancer [93,94,95]. It is quite evident from various studies that DOX has the potential to effectively inhibit glioma cell growth in vitro as well as in vivo [96,97]. Doxorubicin and other anthracyclines are also associated with severe side effects, with cardiotoxicity being the most reported [27], along with significant toxicity to the kidney [98], liver [99], and brain [100]. The mechanism of action of DOX is well known, and it exerts its effects via the inhibition of topoisomerases II, intercalation of DNA molecules in target cells, abrogation of mitochondrial function, generation of ROS, and oxidative damage to cellular components. Despite its efficacy, there are only limited survival benefits for glioblastoma patients undergoing DOX chemotherapy, mainly due to its poor ability to penetrate the blood-brain barrier. The aforementioned factors compel us to look for a better, safer, and more effective alternative formulation to deliver these drugs across the BBB to target glioblastoma. We have attempted to provide a description of doxorubicin carrying liposomes in this section and such studies have also been summarized in the Table 2.

#### 3.2.1. In Vivo Studies

In a study by Gaillard, Pieter J., et al. [101], doxorubicin-encapsulating glutathione PEGylated liposomes (2B3-101) were developed as a new strategy against glioblastoma. The study reported a temperature-, time-, and concentration-dependent uptake of 2B3-101 by an in vitro BBB model consisting of human brain capillary endothelial cells. The in vivo experiments of 2B3-101 in athymic FVB mice tumor models showed an enhanced circulation time, enhanced brain delivery, and enhanced brain retention even after 4 days of administration. The authors also reported that the addition of glutathione as a targeting moiety improved the therapeutic potential of encapsulated doxorubicin in PEGylated liposomes, resulting in a more enhanced inhibition of tumor growth compared to PEGylated liposomes as a control. This was further assured by increasing the dosing frequency from a weekly single dose of 5 mg/kg to a bi-weekly dose. The increase in median survival time, as well as complete tumor regression in mice receiving the treatment, was also reported in this study. A minor drawback of this study was the use of a tumor model that has a comparatively leaky BBB and the failure to establish any dose-dependent toxicity while explaining tumor progression as a cause of mortality in experimental models. Despite the above, these results demonstrated the potential usefulness of targeted liposomal formulations, such as 2B3–101, in improving drug delivery to brain tumors and enhancing treatment efficacy. They provided a promising foundation for future investigations and clinical trials to develop effective liposomal-based therapies for brain cancer patients.

The effect of liposomes encapsulating doxorubicin (Lipo-Dox) as the payload against glioblastoma was investigated by a focused ultrasound (FUS)-mediated disruption of the BBB in a study conducted by Aryal et al. [102]. They tested the effect of FUS and Lipo-Dox in 9L glioma male Sprague–Dawley rat models receiving 3-weekly sessions. Out of 40 experimental animals used in the study, animals administered with liposomal DOX via FUS (N = 8) were reported to have a significant increase in median survival time (*p* < 0.001) in comparison to animals subjected to FUS only (N = 8), doxorubicin only (N = 6), and placebo (N = 7). The 3-weekly chemotherapy of liposomal doxorubicin via focused ultrasound resulted in a 100% change in median survival time compared with controls (without any drug) and a 72% improvement in survival duration in contrast to chemotherapy treatment alone (no FUS, liposomal doxorubicin alone). The authors also reported several side effects associated with this treatment modality, including impaired activity in experimental rats with little or no tumor (owing to side effects from an aggressive treatment regimen) [100]. Furthermore, extensive intratumoral hemorrhage was observed in one of the animals, probably due to the toxicity of doxorubicin formulations [100]. Regardless of the multiple limitations mentioned in this study, there are many favorable takeaways from the findings that can promote the use of this drug delivery method for glioblastoma and may prove to be effective in combination with other treatment regimens for a better cure. Further validation and evaluation studies, including pre-clinical/clinical trials in this regard, may be beneficial to establish such a treatment methodology.

Further specific targeting against glioblastoma was attempted by Lu et al. [103]. The authors formulated a pH-sensitive liposomal model to co-deliver doxorubicin and lonidilamine (LND). The pH-sensitive multi-targeted liposomal assembly, modified with *p*-hydroxybenzoic acid (p-HA) and triphenylphosphonium (TPP), was synthesized to carry a dual payload of doxorubicin and lonidilamine. Their work demonstrated that liposomes possessed excellent pharmacokinetics with the potential to cross the BBB, escaping the cellular endosomal/lysosomal system and delivering the cargo by specifically binding to tumor cells and releasing the drug in a pH-sensitive manner.

**Table 2:** Summary of liposomes in the delivery of doxorubicin against glioblastoma.

Surface Functionalization	Liposomal Payload	Route of Administration	Outcomes	Reference
Glutathione PEGylated liposomes	Doxorubicin	Cell culture and Intravenous	Enhanced Time and temperature-dependent release in BBB model in vitro. Strong inhibition of tumor growth in vivo. Improved survival.	[101]
Liposomal DOX via FUS-mediated delivery	Doxorubicin	Intravenous	Enhanced survival time in vivo. Complete tumor reduction in vivo.	[102]
Transferrin (Tf) and penetratin functionalized liposomes	Doxorubicin and erlotinib	Intravenous and Cell culture	Enhanced penetration in vitro and in vivo. Reduction of tumor volume.	[104]
p-HA and TPP functionalized liposome	Doxorubicin and lonidilamine	Intravenous	Strong inhibition of cancer cell migration and proliferation in vitro. Enhanced drug transport across the BBB, damage to mitochondrial function, and tumor reduction via necroptosis.	[103]
PEGylated liposomes	Doxorubicin-carboplatin	Intravenous	Enhanced cytotoxicity and ROS generation, enhanced drug loading, and drug release. Enhanced the survival time of animal models by 23.1%.	[105]
SS31-functionalized liposomes	Doxorubicin	Intravenous	Increased Uptake-dependent cytotoxicity in vitro, enhanced uptake via the BBB, mitochondrial damage, and suppression of tumor growth.	[106]

The results also demonstrated the enhanced synergistic anti-glioblastoma impact of DOX and LND via promoting necroptosis, obstructing tumor growth by inhibiting glioma cell proliferation, migration, and invasion, with significant damage to mitochondrial function, leading to decreased ATP generation. The results of this study were quite significant in prolonging survival time and inhibiting the metastatic spread of tumors in vivo. This study was also significant in showing the remarkable advantages associated with liposomal dual delivery in comparison to mono-therapy of doxorubicin alone in tackling glioblastoma.

Doxorubicin in combination with carboplatin has been delivered via PEGylated liposomes against glioblastoma by Ghaferi and colleagues [105]. The authors synthesized the assembly with a size range of 202–222 nm and a drug loading efficiency of 10.65%, and a controlled drug release efficiency was reported to be 56.25% of the loaded drugs after 52 h. PEGylated liposomes encapsulating Doxo-carbo (doxorubicin-carboplatin) were reported to enhance cytotoxicity and ROS generation by 1.5-fold and 1.3-fold, respectively, in comparison to non-PEGylated doxorubicin-carboplatin, which enhanced the survival time of animal models by 23.1% (39 days). Additionally, the doxorubicin-carboplatin-carrying PEGylated liposomes were found to have fewer side effects than other combinations used in their study, with strong potential to reduce weight loss in an experimental model. This was a new combination of doxorubicin and carboplatin delivered via liposomes.

Cen et al. [106] developed a dual-targeting liposome for doxorubicin delivery by modifying the DOX-encapsulating liposomes with SS31, a small mitochondria-targeting and BBB-penetrating peptide. The authors reported enhanced drug encapsulation and drug loading, leading to a higher accumulation of the drug inside the target. The in vitro studies reported enhanced uptake-dependent cytotoxicity leading to cell death. The experimental data further showed the enhanced ability of SS31 peptide-modified liposomes to cross the BBB and be taken up by the cells of the glioma and cerebral microvascular endothelium. The in vivo data from nude mice also demonstrated similar results, with an enhanced potential in mediating mitochondrial damage and inhibiting tumor growth with reduced side effects. The impact of dual-targeting with SS31 peptide was quite evident in its ability to cross the BBB and target mitochondria. Although these results are quite promising, low drug-loading efficiency was a concern in this particular study.

#### 3.2.2. In Vitro Studies

Liposomes have also been dual-functionalized to carry doxorubicin and erlotinib as a payload in combination. Liposomal encapsulation of doxorubicin has also been developed in combination with erlotinib. Lakadwala et al. [104] functionalized the liposomes with double surface modifications using transferrin (Tf) and penetratin to encapsulate a dual payload of doxorubicin and erlotinib. While modification with transferrin promotes receptor-mediated endocytosis across the BBB, penetratin is a short cell-penetrating peptide that enhances cell penetration. In vitro biocompatibility, cytotoxicity, hemocompatibility, tumor reduction potential, and ability to cross endothelial barriers were studied in an in vitro tumor model. The biodistribution profiles of functionalized liposomes containing doxorubicin and erlotinib demonstrated more than 12- and 3.3-fold enhancements in the concentration of the respective drugs, making it significantly higher compared to the administration of free drugs (*p* < 0.05). The liposomal assembly was also found to be highly efficient in the reduction of tumor volume, with strong potential for inhibiting tumors by 90% compared to controls.

Doxorubicin-carrying multifunctional liposomes (Mf-LIP) were synthesized from cholesterol/sphingomyelin/PEG and dual-functionalized via mApoE and chlorotoxin peptide to study the role of tunneling nanotubes (TnTs) in the transport of drug–liposome conjugates as a drug delivery channel between glioblastoma cells and normal human astrocytes (NHAs) [59]. This study showed that the cell-to-cell transport of Mf-LIP was more pronounced between U87-MG cells in comparison to the transfer of Mf-LIP between healthy astrocytes. Interestingly, inter-U87-MG transport of Mf-LIP through TnTs was found to be highly preferential compared to NHAs. This work sheds light on the role of TnTs as drug delivery channels in glioblastoma, which may pave the way for more directed approaches to chemotherapy against infiltrating tumor cells in the brain parenchyma.

### 3.3. Liposomes for Nucleic Acid/Gene Delivery in Glioblastoma

**Figure 3:**
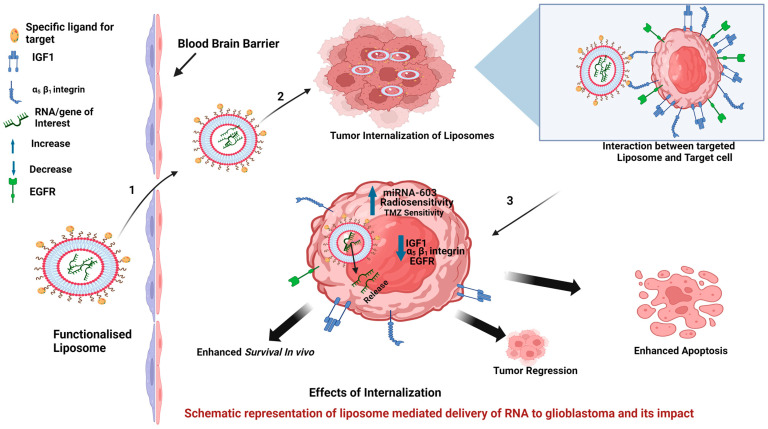
Targeted liposome-mediated delivery of RNA in glioblastoma with subsequent effects on tumorigenesis, expression genes and overexpressed receptors. (1) Penetration of BBB by functionalized liposome. (2) Interaction of liposome with tumor cells within the TME, and (3) Interaction produces downstream effects such as enhanced apoptosis, reduction of tumor volume and increase in the overall survival of the experimental animal.

Due to difficulties and limitations imposed by current glioblastoma therapeutics, nucleic acids are also being explored as potent therapeutic alternatives against glioblastoma. The area of nucleic acid-based therapy is still evolving with strong promise for effective therapeutic intervention against glioblastoma. As a fact, small molecule-based drugs/therapies are generally aimed at proteins or receptor proteins in the target cell and produce desired but temporary therapeutic response, while genetic material- or nucleic acid-based therapies can provide more directed and prolonged therapeutic effects [107]. Nucleic acid-based therapeutics open the way for precision medicines as they are technically powerful and permit a more accurate modulation (upregulation/downregulation) of disease-associated genes [108]. A typical representation of liposomes carrying RNA or genes of interest is shown in Figure 3, for better understanding. To accomplish this, a variety of nucleic acids are being used. To induce a specific gene of interest (GOI), DNA and RNA are most widely used whereas small interfering RNA (siRNA) and micro RNA (miRNA) are used for specific target gene silencing. As a convention, many nucleic acid therapeutics such as siRNA, miRNA, messenger RNA (mRNA), plasmid DNA, antisense oligonucleotides(ASOs), small hairpin RNA (shRNA), and CRISPR/Cas9 have been assessed for their potential as therapeutic agents [109,110].

These nucleic acid therapeutics achieve their therapeutic goal either by the up/down-regulation of target genes, gene knockouts, or gene editing [108]. The additional advantage of these therapeutics is their ease of manipulation/modifications to provide precision-based patient-specific therapies. Such manipulations in nucleic acid sequences can be used to encode specific genes playing a significant role in a particular pathway or even an oncogene to target so-called untreatable tumors [111]. Moreover, specific nucleic acid therapies can target genes involved in oncogenic proliferation, oncogenesis, and apoptotic pathways [44]. Additionally, nucleic acids are also being explored for diagnostic applications in vivo [108]. Despite numerous promises in theranostics for glioblastoma, the delivery of these potential therapeutics is challenging due to degradation by nucleases and unfavorable physiochemical properties. Thus, it is highly imperative to devise a safe, potent, and more directed method for delivery by protecting the nucleic acids from degradation. Keeping this in mind, liposomes emerge as one of the favorable delivery vehicles for nucleic acid delivery in glioblastoma. Here, in the section below, we have presented a detailed account of various liposomes used in the delivery of nucleic acids and the same has been summarized in the Table 3.

Nilmary et al. [112] developed spherical nucleic acids (SNAs) by functionalizing gold NPs and oligonucleotide miRNA inhibitors to target dysregulated microRNA (miRNA). Liposomal constructs modified with apolipoprotein E and rabies virus glycoprotein were used to encapsulate gold-functionalized SNAs for delivery into glioblastoma cells in vitro and glioblastoma tumors in vivo. They reported that the systemic delivery of SNAs into syngeneic mice was enhanced by using ApoE or RVG peptide liposomes as a delivery vehicle and successfully inhibited the expression of the target, miRNA-92b. Furthermore, a higher accumulation of SNAs delivered via ApoE-modified liposomes was also reported in this study. Although this study had no explanation regarding higher accumulation levels of liposome-SNAs in brain tissue and also did not focus on loading and release efficiencies of SNAs via liposomes, it still shows the potential of functionalized liposomes for targeted deliveries of RNAs in glioblastoma.

In a novel therapeutic approach, liposomes as a delivery vehicle were utilized by Lozada et al. [113] to deliver miR-143 oligonucleotide inhibitor (miR-143-inh). They revealed that it selectively targets SLC30A8, a protein associated with glucose metabolism in glioblastoma cells, resulting in cell cycle arrest, decreased cell proliferation, and enhanced apoptosis. Additionally, repeated injections of the liposome-miR-143-inh assembly into glioblastoma tumor-bearing experimental mice inhibited tumor growth compared to controls.

Targeted liposomes have also been used to assess the role of miRNA-603 in combination with polyethylenimine (PEI) in another study [114]. miRNA-603 is a regulatory RNA involved in the suppression of radiation resistance in glioblastoma via the downregulation of IGF1 signaling. This group used PEG and fibronectin mimetic peptide (PRb) to functionalize and specifically target the receptor α5β1. integrin, which is overexpressed in glioblastoma. This targeted delivery resulted in 22-fold increase in miRNA-603 levels in glioblastoma, with a transient decrease in the expression level of insulin-like growth factor 1 and its receptor (IGFR1). This treatment also resulted in enhanced sensitivity of glioblastoma cells toward ionizing radiation, which may open a way toward further improvement in the standard care for the treatment of glioblastoma.

In another targeted approach, a glioblastoma-specific chlorotoxin was utilized as a targeting agent, and the resulting liposomal assembly was used to encapsulate and deliver antisense oligonucleotides (ASOs) or small interfering RNAs (siRNAs) [115]. This study demonstrated enhanced internalization of chlorotoxin-functionalized liposomes in glioblastoma cells, resulting in enhanced delivery and efficacy of the payload. The above nano-formulation caused miR-21-mediated gene silencing in human U87 MG and mouse glioma GL261 cells, resulting in enhanced tumor suppression and decreased cell proliferation through the activation of PTEN and PDCD4, as well as the activation of the caspase-3/7 pathway. This study also shows that the cytotoxic efficacy of the antiangiogenic drug, sunitinib, a strong inhibitor of receptor tyrosine kinase, was also enhanced when delivered with CTX-conjugated, anti-miRNA-loaded liposomes.

Dual-functionalized liposomes with chlorotoxin and transferrin receptor-specific monoclonal antibody OX26 were used by Yue et al. [116] for the targeted delivery of plasmid DNA in glioblastoma. Dual-functionalized liposomes encapsulating plasmid DNAs of choice were shown to have noteworthy cell transfection ability and enhanced translocation across the BBB through selective glioblastoma targeting. These nanoparticle assemblies demonstrated significant cytotoxic and anticancer activities in vitro and high therapeutic potential by reducing tumor volume and increasing mean survival time in tumor-bearing rat models in vivo.

A more directed combination therapy approach was used by Yang et al. [117], where they dual-functionalized liposomes using angiopep-2 and tLyp-1 for the targeted delivery of vascular endothelial growth factor siRNA (VEGF-siRNA) along with docetaxel (DTX). The dual peptide modification enhanced internalization and tissue penetration of the liposomal assembly through receptor-mediated endocytosis in U87 cells and an in vivo mouse tumor model, thereby exerting strong gene-silencing, antiproliferative, and apoptotic activity against malignant cells and tumors.

siRNA for targeted glioblastoma therapy has also been delivered via an aptamer-like peptide (aptide)-functionalized liposomes [118]. The authors showed that the resulting combination of siRNA-encapsulating, aptide-modified liposomes exhibited specific targeting toward extra-domain B of fibronectin (EDB), enhanced uptake in U251 glioma cell lines, and prolonged circulation in vivo in a tumor-bearing mouse model. EDB-targeted, siRNA-carrying liposomes were found to be highly efficient in target gene silencing and anti-proliferative activity in U251 cell lines, as well as inhibiting tumor growth and promoting apoptosis in a xenograft tumor model

To exploit the predominant hypoxic environment in the TME, Liu et al. [119] synthesized hypoxia-responsive malate dehydrogenase-functionalized ionizable liposomes for in vitro and in vivo delivery of polo-like kinase 1 siRNA as nucleic acid therapeutics against glioblastoma. These liposomes were found to be highly efficient in targeted gene silencing, and induction of apoptosis in the treated cells along with tumor growth reduction when tested in a tumor-bearing glioblastoma mouse model.

STAT3 siRNA has been co-delivered with WP1066, a small-molecule inhibitor of the Janus Kinase/STAT pathway, by utilizing functionalized liposomes specific for the α5β1 integrin receptor [120]. Co-delivery of these payloads with α5β1 integrin receptor-targeting liposomes enabled high uptake and internalization within the targeted sites, resulting in a more profound effect. The data showed that the combination was highly cytotoxic and induced apoptosis against GL261 cell lines in comparison to untreated groups and controls; however, no significant cytotoxicity was observed in non-cancerous and CHO cell lines. The authors also reported strong tumor growth inhibition in an orthotopic mouse tumor model with an approximately 350% increase in overall survival in comparison to the untreated glioblastoma tumor-bearing mouse model in vivo

Golgi phosphoprotein 3 (GOLPH3) is a gene product of the *5p13* gene, which has been correlated with glioma progression and shorter survival time [121] and serves as a useful prognostic marker. To target GOLPH3 as a therapeutic target for glioblastoma treatment, Yuan et al. used angiotensin-2 (LRP-1-specific ligand)-functionalized cationic liposomes for the encapsulation and targeted delivery of GOLPH3-siRNA [122]. The authors reported an enhanced internalization of functionalized liposomes into the target cells with enhanced cytotoxic and anti-proliferative activity. Additionally, the in vivo biodistribution studies demonstrated that the angiotensin-cationic liposomes efficiently crossed the BBB, delivered GOLPH3-siRNA at the tumor site, and enhanced overall survival by inhibiting the tumor growth in tumor-bearing nude mice.

Epidermal growth factor receptor (EGFR) overexpression is one of the hallmarks of glioblastoma and can be linked to rapid proliferation and tissue invasion. Angiopeptin-2-modified cationic PLGA liposomes have also been used to co-deliver GOLPH3 siRNA and Gefitinib for synergistic silencing of GOLPH3 mRNA and downregulation of EGFRs by Ye et al. [123]. In vitro and in vivo studies by this group showed that angiopep-2-functionalized liposomes were internalized well and could cross the BBB to deliver the Gefitinib-siGOLPH3 combined payload into the target. This combination of therapeutics mediated the degradation of EGFR via the inhibition of tyrosine kinase signaling and downregulation of GOLPH3 expression, resulting in overall glioma growth inhibition and enhanced survival.

The variable region single-chain fragment from anti-human transferrin receptor monoclonal antibody (TfRscFv) has been used to functionalize cationic liposomes for the delivery of anti-MALAT1 gene [124]. The metastasis-associated lung adenocarcinoma transcript 1 (*MALAT1*) is a long non-coding RNA (lncRNA) implicated in cancer progression and metastasis in various cancers, including glioblastoma. This study showed the efficiency of the liposomal nanocomplex in cancer stem cell targeting and downregulation of *MALAT1* by anti-MALAT1-siRNA, leading to decreased growth, stemness, and the migration of glioblastoma cells. The results also exhibit improved sensitivity of glioblastoma cells to temozolomide. Liposome-mediated *MALAT1* silencing followed by subsequent TMZ treatment demonstrated moderate but statistically significant tumor reduction coupled with overall survival benefits in vivo in moues tumor models.

Liu et al. [125] utilized an environmentally self-adaptive liposomal assembly to achieve CD163 downregulation and co-delivery of the chemotherapeutic drug doxorubicin. CD163 is normally expressed in glioblastoma stem cells and regulates the proliferation and self-renewal of glioma by impacting the CD163/AKT/GSK3β/β-catenin pathway. Here, the authors used shCD163, an inhibitor of CD163, for the suppression of CD163 along with inhibition of cell proliferation, stemness, and tumor progression. The liposomal nanoformulation effectively penetrated and delivered the payload across the BBB. Additionally, the inclusion of doxorubicin as an extra payload enabled enhanced cytotoxicity, tumor reduction, and induction of apoptosis in vivo.

Thermosensitive cationic magnetic liposomes were used by Lu et al. [126] to deliver *SLP2 shRNA* plasmids in combination with the anticancer drug irinotecan (CTP-11) against glioblastoma both in vitro and in vivo. The authors achieved an AMF-regulated, temperature-dependent three-fold increase in drug release from 18% to 59% at physiological pH by changing the temperature from 37 to 43 °C.

**Table 3:** Summary of liposomes based nucleic acid therapies against glioblastoma.

Surface Functionalization	Liposomal Payload	Route of Administration	Outcomes	Reference
ApoE and glycoprotein-modified liposomes	Spherical nucleic acids	Intravenous	Higher accumulation of SNAs, inhibition of target, miRNA-92b expression in vivo and in vitro.	[112]
Liposomes	miR-143 oligonucleotide inhibitor	Intravenous	Cell cycle arrest, decreased cell proliferation, and enhanced apoptosis and inhibition of tumor growth.	[113]
PRb functionalized liposomes	miRNA 603 with polyethyleneimine (PEI)	Intravenous	Downregulation of IGFR1, enhanced sensitivity to ionizing radiation, and suppression of radiation.	[114]
Chlorotoxin modified liposomes	siRNAs	Intravenous	Enhanced tumor suppression and decreased cell proliferation in vivo and in vitro.	[115]
Liposomes modified with Chlorotoxin and OX26	Plasmid DNA	Intravenous	Enhanced cytotoxic and anti-cancer activities in vitro, and highly enhanced reduction of tumor volume and mean survival time in vivo.	[116]
Angiopep-2 and tLyp-1 modified liposomes	VEFG-siRNA with docetaxel	Intravenous	Strong gene silencing, antiproliferative activity, and enhanced apoptosis in vitro and in vivo.	[117]
Aptide functionalized liposomes	siRNA	Intravenous	Gene silencing, enhanced apoptosis in vitro and in vivo, and inhibition of tumor growth in vivo.	[118]
Malate dehydrogenase functionalized ionizable liposomes	polo-like kinase 1 siRNA	Intravenous	Targeted gene silencing and induction of apoptosis and tumor growth reduction.	[119]
α5β1 integrin receptor targeting liposomes	STAT3siRNA with WP1066	Intravenous	Increased cytotoxicity and apoptosis induction in vitro and enhanced survival and inhibition of tumor growth in vivo.	[120]
Angiotensin-2 functionalized cationic liposomes	GOLPH3-siRNA	Intravenous	Enhanced cytotoxic and anti-proliferative activity in vitro and enhanced overall survival in vivo.	[122]
TfRscFv-functionalized liposomes	anti-MALAT1-siRNA	Intravenous	Decreased growth, stemness, and migration of glioblastoma cells. Tumor reduction and overall survival in vivo.	[124]
Self-adaptive liposomes	shCD163 and doxorubicin	Intravenous	Inhibition of cell proliferation, stemness, and tumor progression and enhanced payload delivery across the BBB.	[125]
Thermosensitive cationic magnetic liposomes	SLP2 shRNA	Intravenous	Gene silencing, and more than 50% reduction in the in vitro migration of U87 cells. Reduction of tumor volume and increase in overall survival.	[126]
PEGylated Liposomes	OMIs	Intraperitoneal	Decreased proliferation, migration, invasiveness, and induction of apoptosis in vitro. A decrease in tumor volume and improved survival in vivo.	[127]

The researchers reported a near 100% uptake of the *SLP2 shRNA* gene into target U87 cells via liposomal delivery, resulting in *SLP2* gene silencing and a more than 50% reduction in the in vitro migration of U87 cells. Further in vivo studies in nude mice implanted with U87 xenografts demonstrated a significant reduction in tumor growth and an increase in overall survival. Although this study is promising, there is still a need to assess the impact of using an alternating magnetic field and magnetic NPs by conducting further safety and efficacy studies in animal models, as well as their ability to induce cytotoxicity, ROS generation, mitochondrial damage, and apoptosis in non-cancerous cells as well as in other glioblastoma cell lines.

Liposomal delivery of nucleic acid has also been attempted to study the role of miR-92b, a micro RNA overexpressed in various cancers, including glioblastoma. Nilmary et al. [127] used liposome-encapsulated oligonucleotide microRNA inhibitors (OMIs) for the inhibition of miR-92b. Their data show that inhibition of miR-92b resulted in decreased growth and proliferation, suppressed migration and invasiveness, and induction of apoptosis in T98G, U87, and A172 human glioblastoma cell lines, where the overexpression of the same gene showed an adverse effect. In vivo studies involving subcutaneous mouse tumor models showed a decrease in tumor volume, along with an improved survival rate from the untreated group. Further studies involving different tumor models to analyze and validate the potential of this methodology may pave the way for the development of a more effective miRNA-based therapy for glioblastoma.

## 4. Liposomes in Immunotherapy of Glioblastoma

Immunotherapy involves stimulating a patient’s immune system to target cancer cells. Immune responses in glioblastoma are very weak and challenging due to the presence of the BBB and immunosuppression [128,129]. The presence of the BBB makes the brain an immunologically privileged site; additionally, there is a localized immunosuppression within the TME due to the secretion of various growth factors, cytokines, and chemokines by glioblastoma cells. The typical immunotherapy approaches in glioblastoma include immune checkpoint inhibitors, T-cell-based therapies, and vaccines [130]. Due to the highly immune-suppressive TME and tumor heterogeneity, there are unfortunately no approved immunotherapies or liposome-based immunotherapies against glioblastoma. Liposomes, due to their ability to be functionalized with antibodies or ligands of the choice, along with their ability to carry specific payload, are being considered as a potential tool against glioblastoma immunotherapy.

PI-3065/7DW8-5 encapsulating liposomes were developed by Zhang et al. to enhance the efficiency of the chimeric antigen receptor (CAR)T-cell therapy [131]. The dual payload containing the inhibitor of the PI3K pathway and activator of NK T cells was used to overcome the localized immune suppression in the TME and induce the immune cells functions. Kim et al. used liposome-based nanomedicine SGT-53 to restore p53 function [132]. The results demonstrated that the p53 gene function restoration resulted in enhanced immunogenic changes, antitumor immunity, and significant reversal of tumor-induced immunosuppression in the TME. Sayour et al. utilized cationic liposomes for the intravenous delivery of whole tumor mRNA in a murine model [133]. The results demonstrated that liposomal mRNA activated dendritic cells, activated T-cell populations, and enhanced overall survival. The authors also showed the safety of RNA nanoparticles as systemic therapy in a spontaneous canine glioma model.

**Figure 4:**
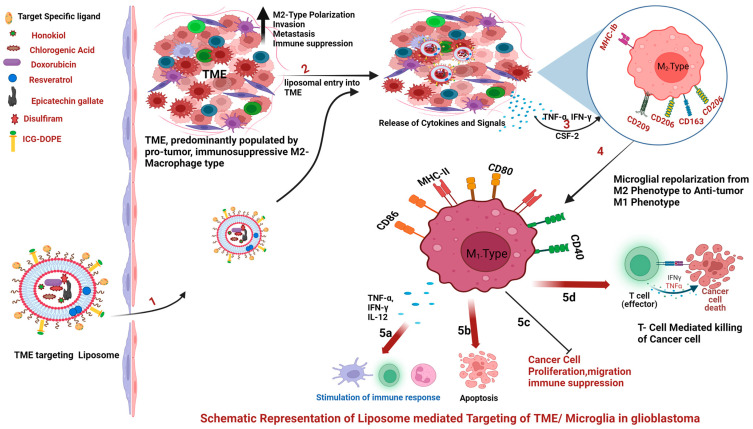
Functionalized liposome-based TME/microglia-specific targeting approaches in glioblastoma. Step 1. Penetration of BBB by targeted liposome, 2. Entry of liposome into TME after BBB penetration and interaction with the target cells, 3. The release of cytokines and other immune effectors specific to M2-type macrophages, 4. Repolarization of microglia from M2-type to anti-tumor M1 type microglia under various factors and 5. Repolarized anti-tumor M1-type microglia stimulate the immune response (5a), induce apoptosis (5b), inhibit the proliferation, and migration of cancer cells and immune suppression (5c) and promote T-cell mediated destruction of cancer cells.

Microglia are specialized macrophages residing in the CNS. Microglial cells, or brain-specific macrophages, are responsible for an effective immune response in the CNS, functioning as major effector cells of the CNS immune system [134]. Microglia, along with tumor-associated macrophages (TAMs), constitute approximately half of the tumor mass as living cells in the TME, thereby indicating their strong role in immune regulation and combat against glioblastoma [135]. Once the macrophages are recruited or infiltrate the glioblastoma TME via a relatively leaky BBB, the TME educates and trains them to secrete different types of regulatory molecules, including cytokines, chemokines, and small extracellular vesicles required to enhance the progression of glioblastoma [136]. The mechanism of the TME and microglial targeting via specialized liposome-based approaches is depicted in Figure 4. There are increasing scientific validations showing the role of TAMs in the progression and promotion of glioma growth, metastasis, and invasiveness [137]. Tumor-associated macrophages and microglia contribute immensely to generating an immunosuppressive TME in glioblastoma, thereby favoring tumor growth, the suppression of immune response, and resistance to chemotherapeutic approaches. Therefore, it is highly imperative to combat and modulate the role of TAMs in tackling glioblastoma progression, along with the improvements in the current therapeutic strategies.

Amongst the early research, targeting microglia via liposomes was achieved by Markovic et al. [138]. The researchers studied the role of microglia in glioblastoma progression and utilized clodronate-encapsulating liposomes for targeted depletion of microglia in cultured brain slice models. Clodronate is a non-nitrogen-containing, first-generation bisphosphonate that inhibits osteoclast activity and has been used in bone metastases and breast cancer [139]. Bisphosphonates like clodronate can deplete TAMs, induce apoptosis, and inhibit cell adhesion [140]. The results demonstrate that clodronate liposomes are efficient in depleting the population of microglia in cultured models, thereby decreasing the invasiveness of glioma.

A recent study by Zhu et al. [141] used a conjugate of pexidartinib (PLX)-encapsulating liposomes and CAR-T cells as an effective combination approach against glioblastoma to re-educate tumor-associated microglia and macrophages (TAMs) towards an anti-tumorigenic and tumor regressive phenotype called M_1_-type macrophage. The authors successfully demonstrated the ability of the conjugate to cross the BBB as well as its prolonged circulation in a glioblastoma mouse model. TAMs-specific targeting and uptake of liposomal assembly resulted in TAMs re-education and their class switching to the M_1_-type macrophage phenotype, thereby enhancing the immune augmentation and antitumor function of CAR-T cells. This further resulted in the complete eradication of glioblastoma tumor growth in 60% of the mice models receiving the combination therapy. This study not only resulted in an enhanced overall survival (OS) of more than 50 days but will also pave the way for more accurate liposome-based therapies in targeting microglia via immunotherapies.

In a study by Ye et al. [125], TAMs have also been targeted via mannosylated liposomes (mannose-functionalized) encapsulating a strong immunoregulatory agent, chlorogenic acid (CHA), which has the potential to promote the polarization of immunosuppressive M_2_-type TAMs towards the M_1_-type. Mannosylated liposomes containing chlorogenic acid as a payload exhibited enhanced circulation in vivo, along with desirable stability and enhanced accumulation in tumors through the mannose receptor-assisted targeting of M_2_ TAMs. Additionally, this combination was found to be highly successful in the inhibition of tumor growth in the G422 glioma model by effectively directing the class-switching of immunosuppressive and tumor-promoting M_2_ TAMs to the anti-tumorigenic microglial subtype called M_1_ TAM phenotype.

In an interesting study, the authors utilized liposomes to target microglia by using them as a BBB-penetrating transport vector for the delivery of paclitaxel [142]. The authors demonstrated the use of DPPS (dipalmitoyl phosphatidylserine) on the surface of paclitaxel-carrying liposomes as an “eat me” signal to promote the uptake of the liposome-paclitaxel assembly by microglia without any significant toxicity. This interesting approach of using the microglia as transport vesicles resulted in effective BBB penetration, glioma-specific migration, and effective delivery of the drug to the target. This study is novel in terms of utilizing microglia as the carriers of drug-loaded liposomes for the treatment of glioma and adds a new dimension to microglia-specific liposomal therapies for glioma.

The M2 microglial phenotype has a characteristic arginase-1 (Arg1) activity, which is associated with the induction of tumor development via promoting cell proliferation, immune suppression, tissue remodeling, and angiogenesis [143]. Honokiol (HNK) is a small bisphenolic lignin derived from the cones and bark of *Magnolia officinalis*. It has garnered considerable interest in recent years due to its strong anticancer properties both in vitro and in vivo [144]. Although honokiol has limited bioavailability and aqueous solubility, it therefore fails to exert its full potential against cancer cells. Li et al. encapsulated HNK in a liposomal formulation (Lip-HNK) to enhance the anticancer activity, bioavailability, and its ability to induce microglial polarization towards the M1 phenotype in glioblastoma [145]. The Lip-HNK was found to be highly efficient in promoting M1 polarization via interferon-gamma and LPS. Interestingly, this HNK-encapsulating liposomal assembly was also found to be effective in the inhibition of microglial polarization towards the M2 phenotype by increasing Arg1 expression and decreasing the expression of iNOS mRNA by IL-4 in a dose-dependent manner. This study also demonstrates the potential Lip-HNK in glioblastoma inhibition both in vivo and in vitro via the induction of M1 microglial polarization and the inhibition of M2 polarization of TAMs. The results are indicative of tumor inhibition, probably through the modulation of microglial polarization.

Photodynamic therapy (PDT), used to induce an anti-tumor immune response, is a clinically approved therapeutic procedure that requires minimal invasion [146]. Indocyanine green (ICG) is known to be a strong photosensitizer for PDT but finds limited applicability due to a lack of specific retention in tumors and is vulnerable to rapid clearance via the kidneys [147]. To overcome this limitation and achieve effective photodynamic therapy, Shabita et al. [148] developed a liposomal system called LP-iDOPE by incorporating 1,2-dioleoyl-sn-glycero-3-phosphoethanolamine (DOPE), conjugated with the ICG fluorophore. The injection of LP-iDOPE in the glioma-bearing animal model resulted in a significant reduction of tumor volume and an increase in median survival time. Further histopathological analysis shows induced cell death via necroptosis and the enhanced infiltration of microglia and CD8+ T-lymphocytes with a significant expression of heat shock protein-70. Interestingly, the thermal effect (hyperthermia) at 45 °C or the interleukin-2-mediated immune reaction did not result in any apoptosis induction.

Zheng et al. [149] developed a liposome-based combination therapy by combining liposomes, honokiol, and disulfiram/copper (CDX-LIPO) to target the mammalian target of the rapamycin (mTOR) pathway for the remodeling of tumor metabolism and the immune microenvironment. The results of this study demonstrated the capability of CDX-LIPO in promoting autophagy, tumor cell death, and tumor regression via promoting anti-tumor immunity through the activation of a variety of immune cells, including natural killer cells (NKCs), dendritic cells, and primed T-cells. Additionally, the above combination resulted in the induced polarization of M_1_-tumor-associated macrophages as well as the restoration of altered glucose metabolism.

Kuang et al. [150] synthesized a matrix metalloproteinase 2 (MMP-2)-responsive peptide-liposome (D@ML). The liposomal surface was functionalized with lipoteichoic acid (LTA), which binds to the monocyte surface via the CD14 receptor. D@ML was further used to encapsulate doxorubicin-hydrochloride. Mild radiotherapy was used to promote the release of Dox-HCL at the intracranial glioblastoma site, which resulted in the induction of immunogenic cell death (ICD) of the glioblastomas. Moreover, the secretion of high-mobility group box 1 (HMGB1) and calreticulin (CALR) from tumor cells induced TAMs’ M1-type polarization, dendritic cell (DC) maturation, and CD8-positive T-cell activation, resulting in a prolonged and enhanced immune response.

Mukherjee et al. developed a triple combination liposomal therapy (TrLp) by combining the turmeric component curcumin and two other polyphenols, namely epicatechin gallate and resveratrol, to achieve a more potent and synergistic effect against glioblastoma [151]. In vitro studies demonstrated that this resulting combination induced p53-mediated apoptosis against GL261 mouse glioblastoma cells and GSCs, whereas the same combination was found to be effective in the repolarization of M_2_-tumor-associated microglia (M_2_-type tumor-associated macrophages), thereby further promoting anti-tumor immunity and augmented immune response in vivo. This research shows the significance of herbal medicine-based liposomal formulations in inducing apoptosis and the repolarization of tumor-promoting microglia.

## 5. Clinical Trials

Despite the encouraging pre-clinical studies concerning liposome-based therapeutic approaches in glioblastoma, complexities due to tumor heterogeneity and BBB have imposed difficulties in evaluating the clinical potential of liposomes in clinical setups [152]. We accessed the online platform ClinicalTrials.gov, the US government’s most authentic online resource by the US government, to find out the ongoing and completed clinical trials of liposome-based therapeutics in glioblastoma. The data from the clinical trials are provided in Table 4.

Unfortunately, the clinical application of liposome-based therapeutics in targeted drug delivery against glioblastoma is still in the developmental phase due to a plethora of challenges, including the BBB, BBTB, and intra- as well as inter-tumor heterogeneity. Nevertheless, there are a small number of approved liposomal interventions for clinical use in other cancers, and their potential is being further investigated via multi-phase clinical trials [153]. One of the earlier approved liposomal formulations is Doxil^®^ or Caelyx^®,^ containing liposomal doxorubicin, which is used for multiple cancers, including metastatic breast cancer and ovarian cancer, and is also being used against glioblastoma [154]. Similarly, the other approved liposomal formulations include DaunoXome^®^ for the treatment of pediatric brain tumors [155] and Depocyt^®^ for the treatment of lymphomatous meningitis [156,157].

The therapeutic potential of Doxil^®^ and temozolomide was assessed in 40 glioblastoma patients by Ananda et al. through a Phase 2 clinical trial [156]. The median PFS was recorded to be 13.4 months, while the median progression time was 6.2 months. While a complete response was shown by only one patient, five were recorded with the progression of the disease, and the remaining twenty-eight showed stable disease. The positive outcomes in terms of stable disease symptoms in a significantly higher proportion of the chosen patients highlight the potential of liposome-based doxorubicin in the suppression of tumor growth.

A Phase 1 clinical trial of the liposomal formulation DaunoXome was conducted in 14 patients with pediatric gliomas by Lippens et al. [155]. The positive outcomes were observed in six children with full response in two, one recurrence in 3 months, and a partial response in the remaining three. Of the remaining eight children, two showed stable diseases, and six were reported to have progressive tumor growth. The variation in response, including relapse, stable disease, positive responses, and tumor progression, necessitates further studies to determine the root causes of the response heterogeneity and failures.

The clinical potential of Doxil was also assessed via clinical trials by Marina et al. in children suffering from refractory brain tumors [158]. The study cohort consisted of 22 child patients, of whom only three patients showed a stable response to the treatment, while the remaining 18 were reported to have the worst disease progression. In another clinical trial by Wagner et al., Doxil^®^ and oral topotecan were used to treat children (n = 8) suffering from advanced-grade brain malignancies [159]. Four of the treated children showed stable disease, whereas the other four were reported to have tumor progression. This study further highlights the challenges in effective treatment and obtaining favorable and progression-free survival in pediatric brain tumors via liposomal therapies.

The variation in the results of clinical trials, with some patients showing prolonged and progression-free survival while others showing no response at all, requires larger clinical trials to validate, optimize, and further establish the efficiency of liposomal formulations [160]. Additionally, a proper understanding of effective routes of administration, combination approaches, and further improvements in current liposomal formulations to achieve positive outcomes for patients receiving liposomal formulations for glioblastoma is required.

## 6. Routes of Liposomal Administration

Numerous routes and strategies for liposomal delivery across the BBB have been developed for the effective treatment of CNS disorders. These strategies can be grouped as physiological, pharmacological, and invasive. Intravenous injection for the delivery of liposomes to the CNS appears to be a preferred choice, but it has its consequences in terms of stability and interaction with immune system components [154,161]. In such cases, the interaction of liposomes with organs such as the liver, and spleen, their half-life in the blood circulation, and interaction with the RES are matters of concern as they may impact the overall fate of the administered therapeutics [162].

The choice between the alternative non-invasive routes for the brain delivery of liposomal formulations (nasal, ocular, or oral) largely depends on the need to bypass the BBB, as it poses the biggest challenge in drug delivery to the brain [163].

### 6.1. The Intranasal (IN)/Nose-to-Brain Delivery of Liposomes

The intranasal (IN) delivery of liposomal nano-formulations has been on a rise in recent years in order to seek better BBB penetrating approaches. Duong et al. [164] and Nguyen et al. [165] have presented a detailed account of various studies involving the IN delivery of liposomal formulations as well as the challenges associated with nose-to-brain delivery of liposomal formulations. The choice between the alternative non-invasive routes for brain delivery of liposomal formulation (nasal, ocular, or oral) largely depends on the need to bypass the BBB, as it poses the biggest challenge in drug delivery to the brain [165,166]. The IN delivery of liposomal formulations is a more practical non-invasive method of delivery across the BBB, as it helps to deliver the drug in comparatively larger amounts than other methods. When pharmaceuticals enter through IN delivery, they are delivered to the brain through the direct nose-to-brain transport system within a few minutes of their administration. The primary mechanism of nose-to-brain transport of liposomal nanoformulations by the IN route involves their distribution to the olfactory region and then routing to the extraneuronal pathways along olfactory neurons to reach the brain [165,167].

Lin et al. prepared a liposomal formulation by encapsulating a butylidenephthalide–(2-hydroxypropyl)-β-cyclodextrin complex for brain delivery in an experimental mouse model bearing TMZ-resistant glioblastoma multiforme [168]. The IN delivery of the liposomal formulation resulted in a prolonged survival time of 60 days in comparison to the oral administration of TMZ and liposomes (with survival times of 36 days and 21 days, respectively). Furthermore, the IN delivery resulted in 10-fold higher drug accumulation in the brain than in oral liposomes after 30 min of administration.

The IN route has also been used for the liposomal delivery of nucleic acids. Liposomes encapsulating c-Myc-targeting siRNA for glioma gene therapy were developed by Hu et al. [169]. The liposomes were functionalized with a cell-penetrating peptide called 89WP for better penetrability, and octa-arginine (R8), a basic amino acid-rich cationic peptide, was employed to compact siRNA by electrostatic interaction to generate a stable core and minimize the premature release of siRNA. The authors reported that the liposomal siRNA was taken up through macropinocytosis by escaping lysosomal entrapment and delivered the nucleic acid payload within 4 h of administration. The IN administration of 89WP-modified siRNA-liposomes efficiently delivered the payload to the orthotopic glioma mouse model because of the enhanced permeability of the nasal mucosa, resulting in enhanced apoptosis and prolonged survival time. This liposomal formulation shows the overall significance of IN delivery to the brain.

### 6.2. Oral Delivery of Liposomes

Oral delivery of pharmaceuticals is one of the easiest non-invasive routes of administration. Although it seems to be an ideal choice in cases of chronic illnesses, there are many hurdles to it. The concerns regarding biocompatibility, as well as how membrane permeability and high first-pass metabolic loss, can affect the bioavailability of the drugs [170].

Oral delivery of liposomes is highly complicated and challenging due to the vulnerability of liposomal compositions. The first and foremost challenge is the exposure of liposomes to the extremely acidic pH of the stomach and harsh conditions of the gastrointestinal (GI) tract, as the conventional liposomal contents, such as cholesterol and phospholipids, are susceptible to the prevalent physiological environment, including gastric acid, lipases, and bile salts [76,170]. The pH sensitivity of the liposomal constituents may trigger the release of the payload. Additionally, lipases can disrupt the liposomal structure through the hydrolysis of phospholipid content, whereas the bile salts secreted by liver can damage the liposome structure through the emulsification process [171].

The first challenge emerges when liposomes are ingested and exposed to the harsh environment of the gastrointestinal (GI) tract. Conventional liposomes, which are composed of phospholipids and cholesterol, are found to be highly susceptible to physiological factors such as gastric acid, bile salts, and lipases [172,173]. The second challenge is associated with the ability of liposomes to permeate through the epithelial surfaces of the GI tract, and unfortunately, little is known regarding the mechanism behind the oral absorption of liposomes. It is highly unclear whether the liposomal contents are released and then absorbed subsequently, or whether the liposome as a whole enters epithelial cells of the GI tract [174,175].

Hu et al. [175] investigated the integrity and stability of oral liposomes containing the bile salt glycocholate (SGC-Lip) in simulated gastrointestinal (GI) media and ex vivo GI media from rats, and compared their absorption and metabolism with conventional liposomes (CH-Lip) composed of soybean phosphatidylcholine and cholesterol. The researchers reported that the encapsulated payload retention was higher in the liposomes with glycocholate in simulated gastrointestinal media containing digestive enzymes like pepsin or pancreatin, as well as in ex vivo gastrointestinal media from the experimental rats. The protection properties of liposomes containing glycocholate was one of the mechanisms contributing to the enhanced oral bioavailability of insulin.

To optimize the lipid composition for the stability of oral liposomes, PEGylation was attempted by Li et al. [176]. The authors demonstrated that the PEG coating allowed the liposomes to evade and tolerate the erosive impact of bile salts, thereby preventing the premature release of the payload. Coating with materials like chitosan, proteins, and other polysaccharides has also been reported for better oral delivery [177,178].

The delivery of oral liposomes is still in the naïve stages, and more research is needed to achieve a highly stable and effective approach for the oral delivery of liposomes. For the efficient stabilization of orally administered liposomes, the uptake efficiency through GI epithelial surfaces must be optimized with enhanced stability to prevent premature release due to the harsh physiological conditions mentioned above [179]. Additionally, it is quite urgent to determine the mechanism associated with the absorption of liposomes through the oral route [170,174].

## 7. Toxicity Studies of Liposomal Formulations

Liposomes hold great potential for delivering chemotherapeutics and nucleic acids in various cancers, including glioblastoma. These are being investigated further to provide a safe and efficient mode of drug delivery for glioblastoma by minimizing the dose-associated side effects and maximizing the therapeutic benefits. There has been a significant rise in liposome-based drug delivery approaches due to the aforementioned advantages. The use of liposomes for the encapsulation and delivery of drugs increases the therapeutic index by minimizing their accumulation in various organs and tissues, thereby decreasing systemic toxicity. For example, the liposomal delivery of doxorubicin minimizes the risk of cardiotoxicity and improves the overall therapeutic response through a decrease in the accumulation of drugs in myocardial tissues. Although the main objective of using liposomes for drug delivery is the prevention of chemotherapeutic toxicity and enhanced delivery, the liposomes or liposomal constituents themselves can induce toxic or allergic reactions and induce an immune response [180,181]. The ability of liposomes to alter the secretion of immune effector molecules has also been documented. Various properties of liposomes, including the degree and extent of functionalization, PEGylation, size, and surface charge, affect the level of immunogenicity [182,183,184].

PEGylation is one of the most commonly employed methods, utilizing PEG polymers to covalently modify the surface and enhance the blood dynamics of liposomes. The early reports of PEGylation-induced toxicity and immunogenicity led to significant interest in the research community, which was further supported by PEG-liposome-based COVID-19 vaccines [181,185]. The safety and immunogenicity of PEG have also been studied in detail, and free PEG has been given Generally Recognized as Safe (GRAS) status by the US FDA and is believed to be non-antigenic and immunogenic. Therefore, the role of PEG in immunogenicity is somewhat debatable, as many studies have also shown that the anti-PEG antibodies are generally formed against antigenic determinants found at the junction between PEG and other materials, and not directly against PEG itself [186]. A study conducted by Shiraishi et al. [187] showed the accelerated blood clearance (ABC) phenomenon of polyethylene glycol-1,2-Dioctadecanoyl-sn-glycero-3-phosphoethanolamine (PEG-DSPE) comprised liposomes, whereas the similar quantity of PEG lipid in polymeric micelles did not elucidate any significant immune reactivity and antibody generation.

Cationic liposomes are the preferred choice for the delivery of nucleic acids and for targeting the negatively charged BBB [126], and have been reported to induce toxic effects in various immune cells, including macrophages [184]. The immunogenic potential of cationic liposomes was demonstrated by Takano et al. and Aramaki et al. They correlated the intracellular uptake by the mouse macrophage-like cell line RAW264.7 with the degree of apoptosis and generation of ROS [183,188]. Conversely, the liposomal uptake was reduced by approximately 30–45-fold through the incorporation of 7.5% and 10% 1,2-dipalmitoyl-sn-glycero-3-phosphoethanolamine (DPPE-PEG) into the liposomal formulation. This led to a decline in ROS production [183], liposomal toxicity, and approximately a 20% to 38% increase in cell viability [188]. Kedmi et al. [189] studied various aspects of liposomal toxicity, such as hepatotoxicity and inflammatory responses, using in vivo animal models. They reported a reduction in body weight and a multifold rise in the level of hepatic enzymes in the serum of C57BL/6 mice. A notable (10–20-fold) rise in the level of Th1- and Th17-dependent cytokines was also reported after two hours of injection. In a similar study involving a rat in vivo model, the treatment with DOTAP/CHOL liposomes not only up-regulated the gene responsible for oxidative stress (HMOX1) and oxoguanine glycosylase (OGG1), the enzyme for DNA repair in the liver, but also resulted in enhanced DNA strand breaks in the lung [190].

Liposomal formulations are typically encountered by a system of phagocytic cells called the mononuclear phagocytic system (MPS) or the RES, located at multiple sites, including liver, bone marrow, lymph nodes, and spleen [191,192,193]. Several studies conducted on rodents have reported the accumulation of Doxil primarily within the liver and spleen, rather than in other organs, after intravenous injections [194,195]. Allen et al. employed several compositions of egg PC-CHOL liposomes to analyze the effect of liposomes on the phagocytic ability of the MPS in experimental mice [196]. In comparison to small unilamellar liposomes, the intravenous administration of large multilamellar liposomes in mice resulted in approximately a 50% decrease in the phagocytic index of the liver. No recovery in the phagocytic index was observed before three-weeks after injection. Additional doses of liposomes caused enhanced accumulation and a 2.5-fold increase in the spleen of experimental mice.

Cholesterol is one of the important constituents of liposomes added to enhance the stability and functionality of liposomal structures and adds to the permeability and surface charge. Apart from its role in stability and functionality, its role in toxicity and immunogenicity has also been examined. In a study by Adams et al., 5–10 mg of cholesterol injection caused mild edema, tissue damage, and the loss of neuronal structure in the mouse brain. A Distearoylphosphatidylcholine (DPSC) based liposomal formulation containing 50 mol% of cholesterol decreased liposomal accumulation in the liver of rats and caused up to a three-fold increase in systemic circulation half-life [197]. Additionally, the role of cholesterol content in liposomal structures in decreasing the decline in the immunoglobulin G-mediated response and macrophage uptake has been examined [198]. Liposomes, after their administration, interact with circulating proteins that get adsorbed on the liposomal surface, forming a unique protein corona depending on the liposomal properties. The subsequent immune suppression or augmentation may occur due to this protein corona [199]. Li et al. [199] and Kinsky et al. [200] have also reported the role of liposomes in activating a complement-mediated immune response by interacting with complement proteins and subsequently initiating a complement cascade.

In summary, liposome-mediated toxicity to healthy tissues, coupled with the activation of the immune system, raises important concerns regarding their use, and liposomes themselves can impart new toxicities that are still not fully understood. While liposomes are important drug-delivery vehicles in the delivery of chemotherapeutics, nucleic acids, and vaccines, a thorough assessment of their toxicity and immunogenicity profile is critical for the development of a safe and effective treatment strategy against glioblastoma

## 8. Conclusions and Future Prospects

Glioblastoma is a fatal brain tumor and requires a very aggressive standard of treatment consisting of surgery, radiotherapy, and TMZ-based chemotherapy. The efficacy of current therapeutic approach is limited due to the poor bioavailability of TMZ, high recurrence rate, the presence of the BBB, and tumor heterogeneity. Among the current therapeutic options being explored, the advent of nanomedicine and nanotechnology provides promising and novel drug delivery options against glioblastoma. Recently, liposomes, among the NPs, have emerged as highly promising and potent drug delivery tools to tackle glioblastoma. Liposomes are fatty acid/lipid-based synthetic nanostructures that can be synthesized and functionalized in multiple ways for customized drug delivery applications. They offer several advantages over other drug delivery systems, including their ability to protect drugs from degradation, improve solubility, and provide controlled drug release. Importantly, liposomes can be designed to target specific cells or tissues, making them an attractive option for delivering drugs to brain tumors. The net charge on the target or cellular microenvironment dictates the charge type on liposomes. The exact type of liposomal choice will depend on the area or environment being targeted. The use of cationic liposomes in the delivery of therapeutics to glioblastoma is useful due to the negative charge of the BBB, which may favor an electrostatic interactions resulting in enhanced uptake via adsorption-mediated endocytosis. Additionally, the liposomal surface can be decorated with a variety of targeting moieties or ligands exhibiting high affinity and specificity toward a particular target. For example, the use of transferrin conjugation by Lam et al. [84], glutathione by Gaillard, Pieter J., et al. [101], the use of TfRscFv by Ma et al. [124], angiotensin-2 (an LRP-1-specific ligand) by Yuan et al. [122], and use of angiopep-2 and tLyp-1 by Yang et al. [117] are some mentioned approaches that can be further optimized and assessed for their potential for targeted payload delivery in glioblastoma. There are many methodologies and molecules discussed in this review regarding the modifications/functionalizations of liposomes for customized drug delivery applications; nevertheless, this approach also has considerable drawbacks, especially regarding the change in the orientation of ligands on the liposomal surface. These challenges, therefore, advocate for a very careful and precise methodology for modified liposomes intended to be used in glioblastoma therapy. Irrespective of the concerns, modified and surface-functionalized liposomes have offered new avenues for the minimization of dose-dependent toxicities, enhanced bioavailability, and tackling drug resistance and the development of targeted and precision-based medicine in glioblastoma. Despite the intensive research and development and partial successes, there are still challenges to be addressed in developing liposomal drug delivery for glioblastoma therapy. For example, optimizing liposome formulations to improve drug release and stability, as well as developing effective targeting strategies to enhance tumor specificity, will be important for improving treatment outcomes. Additionally, scaling up production and reducing costs will be important for making these treatments more widely accessible. In conclusion, liposomes have demonstrated great potential as drug delivery systems for glioblastoma therapy.

Considering the potential threats of mortality and rapid recurrence, there is a need for a rapid cure against glioblastoma. The functionalized or targeted liposomal drug carriers could become the first line of therapeutic interventions against glioblastoma, given the proper attention of the research community. Attaching a target-specific ligand to a liposomal surface is highly promising for targeting cancer cells and delivering personalized treatment effectively and safely. Transferrin conjugation on the liposomal surface is an effective strategy for targeting the delivery of drugs across the BBB and increasing its accumulation at the tumor site [84]. Similarly, the approach of dual functionalization of liposomes with transferrin (Tf) and penetratin to encapsulate a dual payload of doxorubicin and erlotinib is also very promising, as transferrin can potentially cross the BBB, whereas penetratin can penetrate the target cell. Developing a multifunctional liposomal vehicle with multiple targeting peptides/ligands can be an effective model for drug delivery in glioblastoma. Targeting the TME and tumor-associated macrophages (TAMs) or microglia via functionalized liposomes could be another potential tool for hunting down glioblastoma via induction of a strong immune response in the TME, as localized immune suppression and M2 polarization of microglia are matters of great concern. Additionally, studies such as the use of mannosylated liposomes for the transport of the immunoregulatory agent CHA [125], the use of Lip-HNK (Liposomes-Honokiol) to promote macrophage activation [145], and the utilization of a triple combination liposomal therapy (TrLp) [151] to induce microglial polarization toward the anti-tumor M1 phenotype and the inhibition of M2 polarization of TAMs are very promising and can play a very crucial role in the development of microglia-based immunotherapies against glioblastoma in the near future.

## Figures and Tables

**Table 1:** Summary of liposomes in the delivery of TMZ against glioblastoma.

Surface Functionalization	Liposomal Payload	Route of Administration	Outcomes	Reference
PEGylated liposomes	TMZ	Intravenous	Enhanced survival and tumor reduction in vivo.	[80]
PEGylated liposomes	TMZ	Intravenous	Prolonged release with enhanced apoptosis in vitro and enhanced blood circulation time with significant tumor reduction in vivo	[82]
Glucose-PAP functionalized liposomes	TMZ	Intravenous and subcutaneous	Enhanced BBB penetration and TMZ accumulation with tumor reduction and prolonged survival in vivo.	[43]
Cationic DOTAP liposomes	TMZ	Intravenous	Effective BBB penetration and drug accumulation in increased apoptosis and drug release in vitro.	[91]
Transferrin-conjugated liposomes	TMZ and bromodomain inhibitor JQ1	Intravenous	effective delivery across the BBB, DNA damage, and enhanced apoptosis in vivo.	[84].
ART-PC (artesunate- phosphatidylcholine) liposome	TMZ	Intravenous	DNA damage via the generation of ROS, tumor reduction, enhanced overall survival.	[57]
Thermosensitive liposomes	Magnetic NPs and TMZ	Cell culture	ROS-mediated pyroptosis, oxidative damage to DNA.	[89]
Liposome–hydrogel composite	TMZ	Cell culture	Enhanced anti-tumor effect against the 3D spheroid glioblastoma model in vitro, and inhibition of tumor growth.	[90]

**Table 4:** Summary of ongoing/completed clinical trials for glioblastoma therapy.

Sponsor Organization	ClinicalTrials.gov ID	Liposomal Payload	Total Number of Patients	Clinical Trial Phase	Status
University of Regensburg	NCT00944801	Doxorubicin, temozolomide	63	I and II	Completed
Medical University of South Carolina	NCT01044966	Cytarabine (DepoCyt), temozolomide	12	I and II	Aborted due to lack of an adequate number of patients
University of California, San Francisco	NCT00734682	CPT-11	34	I	Completed
Plus Therapeutics	NCT01906385	Rhenium186 nanoliposomes (186RNL)	55	I and II	On-going
Emory University	NCT04590664	Visudyne (liposomal verteporfin)	24	I and II	On-going
Northwestern University	NCT05864534	Drug Balstilimab and Botensilimab along with liposomal doxorubicin	25	II	On-going
University of Florida	NCT06389591	RNA vaccine	24	I	On-going
SignPath Pharma, Inc. Utah	NCT05768919	Curcumin	30	I and II	On-going
Université de Sherbrooke	NCT06356883	Intraarterial Carboplatin + Caelyx (liposomal doxorubicin) vs. intraarterial varboplatin + etoposide	120	II	On-going
Plus Therapeutics	NCT05460507	186RNL	40	I	On-going
University of Florida	NCT04573140	mRNA-loaded DOTAP liposome vaccine	52	I	On-going
Institut de cancérologie Strasbourg Europe	NCT06477939	Liposomal transcrocetin (L-TC) with radiotherapy plus temozolomide	554	III	Yet to be started
